# The Extracellular Vesicle–Macrophage Regulatory Axis: A Novel Pathogenesis for Endometriosis

**DOI:** 10.3390/biom13091376

**Published:** 2023-09-12

**Authors:** Xiaoxiao Gao, Han Gao, Wei Shao, Jiaqi Wang, Mingqing Li, Songping Liu

**Affiliations:** 1 Department of Obstetrics and Gynecology, Jinshan Hospital, Fudan University, Shanghai 201508, China; 22211270005@m.fudan.edu.cn (X.G.); gaohan5041551@163.com (H.G.); shaowei@fudan.edu.cn (W.S.); jiaqiwang199904@163.com (J.W.); 2 Laboratory for Reproductive Immunology, Institute Obstetrics and Gynecology, Hospital of Obstetrics and Gynecology, Fudan University, Shanghai 200080, China

**Keywords:** endometriosis, macrophages, extracellular vesicles, pathogenesis, lncRNA, miRNA

## Abstract

Endometriosis (EMs) is a common disease among women whose pathogenesis is still unclear, although there are various hypotheses. Recent studies have considered macrophages the key part of the immune system in developing EMs, inducing inflammation, the growth and invasion of the ectopic endometrium, and angiogenesis. Extracellular vesicles (EVs) as novel intercellular vesicle traffic, can be secreted by many kinds of cells, including macrophages. By carrying long non-coding RNA (lncRNA), microRNA (miRNA), or other molecules, EVs can regulate the biological functions of macrophages in an autocrine and paracrine manner, including ectopic lesion growth, immune dysfunction, angiogenesis, and can further accelerate the progression of EMs. In this review, the interactions between macrophages and EVs for the pathogenesis of EMs are summarized. Notably, the regulatory pathways and molecular mechanisms of EVs secreted by macrophages during EMs are reviewed.

## 1. Introduction

EMs affects 5–10% of women of reproductive age worldwide, and is characterized by the presence of uterine endometrial tissue outside of the normal location, mainly on the pelvic peritoneum, ovaries, rectovaginal septum, and more rarely in the pericardium, pleura, and even the brain [1,2]. Although it is a common, benign, estrogen-dependent, chronic gynecological disorder, it displays some tumor-like phenotypes with high migration and invasion abilities [1,3]. EMs patients often have pelvic pain, infertility, dysmenorrhea, dyspareunia, etc., which involves complex endocrine, immunologic, proinflammatory, and proangiogenic processes [4,5,6,7].

There are many hypotheses for the pathogenesis of EMs, including Sampson retrograde menstruation, coelomic metaplasia, immune theory, and Müllerian remnants [2,8,9,10]. Among these, immune dysregulation stimulated by the presence of endometrial debris on the peritoneum of patients with EMs is involved in the pathogenesis of the disease [11,12,13]. Inflammatory cytokines and immune cells increase the risk of disease development and EMs-related infertility by promoting the survival, growth, invasion, angiogenesis, and immune escape of endometriotic lesions [14,15,16]. There is growing evidence that these immune cells are activated by menstrual debris and subsequently participate in the inflammatory process, and that macrophages are the primary contributor to pro-inflammatory cytokines [17].

Macrophage is an important component of the ectopic microenvironment. Although macrophages play an important role in the homeostasis of the peritoneal environment, they mediate inflammation and facilitate the establishment and maintenance of the disease [17]. Activated peritoneal macrophages secrete inflammatory substances that promote the proliferation, adhesion, and angiogenesis of ectopic endometrial cells, leading to abnormalities in the immune system, local pelvic adhesion, and fibrosis in EMs [18,19,20,21]. Macrophages also meditate autophagic flux to promote the proliferation and migration of endometrial stromal cells (ESCs) through the *CCL20*/*CCR6* axis [22]. Research in vivo has shown that injecting with M1 macrophages leads to a severely disrupted architecture and the suppression of EMs development in a mice model [21]. In contrast, M2 macrophages resulted in the increased growth of lesions [21].

Exosomes are one of the extracellular vesicles’ populations, based on their biogenetic pathway, composition, and physical characteristics such as size or density [23]. There are four major categories of EVs: apoptotic bodies, retrovirus-like vesicles, macrovesicles, and exosomes [23,24]. Among these, exosomes are membraned organelles of approximately 30–200 nm in diameter [25]. The EVs are important in transferring messenger RNAs (mRNAs), miRNAs, and double-strand or genomic DNA [26]. It has been confirmed that EVs can influence cell behavior in an autocrine or paracrine manner, and enhance pro-angiogenic effects, neurogenesis, immune modulation, and endometrial stromal cell invasion [27,28].

The applications of EVs, including the pathogenesis, diagnosis, and treatment of diseases, offer distinct advantages that uniquely position them as research objects [29,30,31,32].

In EMs, the content of exosomes is in different parts, such as blood, peritoneal fluid, and ectopic lesions [33,34,35]. Exosomes from endometrial epithelial cells (EECs) or ESCs of ectopic lesions regulate their invasive and migratory potential [3,36]. Additionally, EVs play an important role in macrophage activation and polarization [37]. Of note, immune cells and exosomes can interact with each other. In turn, exosomes derived from macrophages affect the development of ectopic lesion of EMs via multiple pathways [38].

In this review, therefore, we attempt to outline the generation and function of EVs in EMs, and further elaborate upon the potential roles of the macrophage–exosome axis in the pathogenesis of EMs.

## 2. Abnormal Levels of EVs in EMs

### 2.1. Peripheral Blood

As a physiological fluid, blood plays a crucial role in facilitating cell-to-cell communication and the transportation of EVs to distant cellular targets through the circulatory system [39]. As shown in Table 1, exosomes were observed in the serum of patients with EMs, and specific circulating miRNAs from EVs were considered valuable indicators for the severity of EMs, including serum *miR-26b-5p*, *miR-215-5p*, and, *miR-6795-3p* [40]. Moreover, 24 differentially expressed miRNAs were identified in the serum EVs between control and EMs patients via a miRNA microarray analysis [33]. Among these, two miRNA (*miR-22-3p* and *miR-320a*) were significantly upregulated in serum EVs from EMs patients, suggesting that these miRNAs of serum EVs should be the biomarkers for EMs diagnosis [33]. It is worth noting that further research on their mechanisms is still required.

More importantly, a bioinformatics analysis showed that the differentially expressed miRNAs in serum EVs between control and EMs patients are mainly enriched in the regulation of cell development and metabolism, and involved in the regulation of the MAPK and PI3k-Akt pathways [40]. It has been reported that plasma EVs from EMs patients stimulate the proliferation of endometriotic epithelial and endothelial cells in vitro [41]. Additionally, serum EVs and ectopic endometria significantly release lncRNAs such as lncRNA antisense hypoxia-inducible factor (lncRNA *aHIF*) [42]. The levels of lncRNA *aHIF* in the serum exosomes of patients with EMs showed a significant correlation with the expression of lncRNA *aHIF* in corresponding ectopic endometrial tissue, which suggests that lncRNA *aHIF* could be transported in serum via exosomes [42].

**Table 1:** The sources and expression molecules of EVs in EMs.

Generation	Expression in EVs		Reference
Peripheral blood	miR-26b-5p	↑	Wu et al., 2022 [40]
	miR-215-5p	↓	
	miR-6795-3p	↑	
	miR-320a	↑	Zhang et al., 2020 [33]
	miR-22-3p	↑	
	lncRNA aHIF	↑	Qiu et al., 2019 [42]
Peritoneal fluid	PRDX1, histone H2A type 2-C, ANXA2, ITIH4(fragment), tubulin a-chain	-	Nazri et al., 2020 [35]
Ectopic endometrium	lncRNA aHIF	↑	Qiu et al., 2019 [42]
	miR-15a-5p	↑	Liu et al., 2016 [43]
	miR-301a-3p	↑	Huang et al., 2022 [44]
	miR-214	↑	Wu et al., 2018 [45]
	miR-30c	↓	Zhang et al., 2022 [3]
Immune cells	tRF-Leu-AAG-001	↑	Li et al., 2022 [46]
	miR-22-3p	↑	Zhang et al., 2020 [47]
	Lnc RNA CHL-AS1 miRNA-1908, -130b, -451a, -486-5p, -4488, -432, -342, -425, -505, -6508, -145, -365a, and -365b	↑ ↑	Liu et al., 2021 [38] Chen et al., 2019 [48]

Abbreviations: PRDX1, peroxiredoxin-1; ANXA2, annexin A2; ITIH4, inter-a-trypsin inhibitor heavy chain H4; aHIF, antisense hypoxia-inducible factor. ↑: the increased expression in EVs; ↓: the decreased expression in EVs.

### 2.2. Peritoneal Fluid

EVs are present in peritoneal fluid (PF), indicating their potential involvement in EMs [35]. The concentrations of macromolecules in PF were found to be significantly higher in women with EMs than that in patients without EMs, and these alterations might be involved in regulating the microenvironment of PF [49,50]. In addition, many proteins were also detected in the PF EVs from EMs patients, including peroxiredoxin-1 (PRDX1), histone H2A type 2-C, annexin A2 (ANXA2), inter-a-trypsin inhibitor heavy chain H4 (ITIH4) (fragment), and tubulin a-chain [35]. More interestingly, the concentration of EVs in the PF is closely correlated with disease stage and menstrual cycle [35]. Nazri et al. found that the concentration of PF EVs from patients with I-II EMs was higher than that of III-IV EMs, and the more prominent inflammatory cells in the stage of I-II should be involved in this difference [35].

### 2.3. Ectopic Endometrium

EVs can be isolated from the endometrium, uterine cavity, and pregnant uterus [34,51,52]. Normal EECs-derived exosomal *miR-30c* has been reported to inhibit the invasion and migration of ectopic EECs, thereby attenuating the metastasis of ectopic nodules [3]. Moreover, exosomes isolated from ectopic endometrial tissues have been found to influence the phagocytosis and polarization of macrophages by releasing *miR-301a-3p*, suggesting their roles of EVs from ectopic endometrial tissues in immune regulation [44]. Wu et al. reported that exosomal *miR-214* from ectopic endometrial stromal cells (eESCs) could inhibit ectopic lesions’ fibrosis; however, further in vitro or in vivo studies are necessary to confirm this [45].

It has been reported that ESCs with EMs may induce neurite outgrowth and inhibit neuron apoptosis. Importantly, high levels of EVs from ESCs positively correlate with neurite outgrowth [53]. The blocking of EVs reduces pro-neuro angiogenesis induced by EVs, indicating that EVs play an important role in neuro-angiogenesis in EMs [53]. Qiu et al. demonstrated that enriched lncRNA *aHIF* from ESCs-derived EVs can facilitate the angiogenesis of EMs by activating vascular endothelial growth factor (VEGF)-A, VEGF-D and basic fibroblast growth factor [42,54,55]. In addition, the expression of *miR-15a-5p* in EVs from ovarian ectopic endometrium was downregulated [43]. Further analysis showed that *miR-15a-5p* could suppress the progress of EMs by targeting VEGF-A [43,56]. Therefore, these findings highlight the potential of exosomal miRNAs in modulating key factors involved in the pathogenesis of EMs.

### 2.4. Immune Cells

The development of EMs is closely related to the immune system [57]. In ectopic lesions, immune cell infiltration is greater than in normal lesions [57]. Exosomal tRF-Leu-AAG-001 derived from mast cells in ectopic foci should promote inflammation and angiogenesis, as well as the increases in IL-6, IL-10, IL-1β, and TNF-α [46].

In early-stage and advanced-stage EMs, there were 13 differential miRNAs (*miRNA-1908*, *-130b*, *-451a*, *-486-5p*, *-4488*, *-432*, *-342*, *-425*, *-505*, *-6508*, *-145*, *-365a*, and *-365b*) of EVs in PF myeloid-derived suppressor cells (MDSCs) and regulatory cells (Tregs), and these miRNAs were enriched in the regulation of immune and cell proliferation [48]. These data illustrate that these miRNAs of EVs in PF MDSCs and Tregs should contribute to the peritoneal immunosuppressive microenvironment [48].

Macrophages are more likely to be attracted to the peritoneal environment, which mainly produces PF growth factors and inflammatory mediators [58,59]. EVs released by peritoneal macrophages could deliver *miR-22-3p* and promote the proliferation, migration, and invasion of eESCs by targeting SIRT1 and activating the NF-κB pathway [47]. Moreover, exosomal lncRNA *CHL-AS1* from peritoneal macrophages can also affect the proliferation, migration, invasion, and apoptosis of eESCs [38].

Meanwhile, EVs derived from eESCs could be transferred to macrophages and influence the function of macrophages [47]. Therefore, EVs should be involved in the crosstalk between ESCs and macrophages in EMs. More research is required to confirm the mechanisms of these miRNA molecules, and to examine their clinical applications.

## 3. Regulation of the Immune Microenvironment and Macrophage Function

EVs act as a novel transferrer in various physiological and pathological conditions and EMs. Herein, the regulatory effect of EVs in EMs on macrophage and the immune environment is elaborated upon (Table 2).

### 3.1. Inflammatory

EMs is characterized as a chronic inflammatory disease, prominently displaying an inflammatory response [64,65]. This inflammatory state contributes to the manifestation of endothelial dysfunction, the emergence of EMs-associated pain, and potentially predisposes individuals to carcinogenesis [65,66,67]. EVs play a critical role as carriers of genetic information in the form of RNAs, and serve as mediators of inflammation [68]. Ectopic tissue in the peritoneal cavity is associated with the overproduction of prostaglandins, cytokines, and chemokines [69]. Macrophage-derived miRNAs can be encapsulated within EVs, effectively regulating inflammation and cell signaling [70]. Moreover, EVs have also been reported to engage in pro-inflammatory activity and contribute to the innate immune response by releasing IL-1β, IL-6 and TNF-α during inflammasome activation, thereby fostering an inflammatory environment for EMs development [71,72,73]. Exosomal long non-coding homeobox transcript antisense RNA (lncRNA *HOTAIR*), derived from ESCs, could downregulate the expression of miR-761 and increase histone deacetylase 1(HDAC1), and further activate STAT3-related proinflammatory cytokines [36]. These inflammatory responses induced by EVs promote the progression of EMs and angiogenesis in vivo and in vitro [36].

In an experimental study, *miR-138* was confirmed to exacerbate inflammation in macrophages by intraperitoneally injecting LPS into an animal model, resulting in tissue damage and organ dysfunction [60]. Additionally, *miR-138* possibly secreted by EVs was markedly decreases in EMs mice, and the down-regulation of *miR-138* resulted in the apoptosis and inflammation of uterine endothelial cells, and the activation of VEGF/NF-κB signaling pathway in THP-1 cells [60]. Therefore, exosomal *miR-138* may activate macrophages through the NF-κB pathway, leading to inflammation and promoting the development of EMs. However, further investigation is required to elucidate the precise mechanism between *miR-138* and macrophages in EMs with more clinical studies.

### 3.2. Polarization of Macrophages

Two distinct phenotypes of activated macrophages, commonly referred to as M1 and M2, play a pivotal role in the initiation and development of EMs [74]. M1 macrophages have been found to in situ to predominate the endometrium of patients with EMs compared to healthy controls [75]. The peak of inflammatory M1 macrophage markers early in the development of EMs-like lesions is followed by a transition from classical M1 macrophage activity to an alternate M2 profile, with an increase in the peritoneal Th2 and Treg cell populations, which may further contribute to the formation of an immunosuppressive microenvironment in EMs [76,77,78,79]. Understanding the distinct phenotypes and functions of activated macrophages in EMs provides insights into the immunological and molecular mechanisms underlying the initiation and development of the disease. Targeting these macrophage phenotypes and their associated pathways may offer potential therapeutic strategies for EMs.

In EMs, the MAPK signaling pathway is important for regulating macrophage function and plasticity [80,81]. The elevated expression of *miR-210-3p* was observed in the eutopic endometrium of EMs patients during both the proliferative phase and the secretory phase, as well as in the EVs [52]. Further analysis showed that exosomal *miR-210-3p*, derived from the uterine aspirate fluid, could modulate the MAPK signal pathway by inhibiting the key protein kinase c-Jun N-terminal kinase (JNK), further resulting in an decreased proportion of CD80+ M1 macrophages [52,80].

Infiltrating macrophages contribute to the inflammatory response and tissue remodeling associated with the disease [82]. The macrophage density in the eutopic endometrium and red lesions significantly correlated with microvessel density [83]. Specifically, more M2 macrophages primarily infiltrated the ectopic glands of the lesions [84]. Emerging studies have demonstrated that alternately activated macrophages can infiltrate endometriotic lesions and promote angiogenesis [21]. Endometriotic lesions with a higher density of macrophages are also associated with a significantly increased prevalence of nerve fibers, which may contribute to the experience of pain [85]. A study conducted by Sun et al. proved that in the mice model, more M2 macrophages primarily infiltrated the ectopic glands of the lesions [37]. After injection of EVs from ectopic ESCs, more M2 macrophages primarily infiltrated the ectopic glands of the lesions [37]. Importantly, the volume and weight of ectopic lesions were found to increase proportionally with the number of infiltrating M2 macrophages, thereby accelerating the progression of endometriotic lesions in mice [86]. As an antagonist of the PI3K/AKT signaling pathway, exosomal *miR-301a-3p* from the ectopic endometrium of EMs patients could inhibit PTEN expression and promote the expression of Arg-1 and PI3K to promote the polarization of macrophages to M2 macrophages, as well as cell proliferation [44].

M2 macrophages are widely recognized as a key modulator of fibrogenesis and contribute to high levels of transforming growth factor (TGF)-β and collagen deposition [87,88,89,90]. Notably, exosomal *miR-214-3p* derived from ESCs is one of the down-regulated miRNAs in endometriotic cystic stromal cells [61,91]. Previous studies have demonstrated that *miR-214-3p* enhances M2 macrophage polarization by targeting the glycogen synthase kinase 3 beta(GSK3B), subsequently promoting fibrosis in Ems [62]. Moreover, the level of *miR-214-3p* was decreased in the Evs from ectopic ESCs [45]. Further study showed that the down-regulation of *miR-214* promoted Collagen αI and connective tissue growth factor (CTGF) expression significantly elevated in endometriotic lesions in mice model [45,92]. These findings indicate that Evs should regulate macrophage polarization in endometriotic milium, further accelerating the development of Ems, especially fibrosis.

### 3.3. Macrophage Phagocytosis

The phagocytic activity of peritoneal macrophages is considered crucial in healthy women to eliminate menstrual detritus [93]. The presence of an augmented inflammatory phenotype and reduced phagocytic ability of endometrial macrophages in individuals with Ems aligns with impaired elimination of shed endometrial cells during menstruation, and in maintaining tissue equilibrium [94]. Endometrial cells demonstrate greater resistance to apoptosis and the macrophages in the peritoneal fluid [95]. Compared to the normal human serum EVs group, the phagocytic ability of macrophages to the EVs from ectopic endometrial tissues was decreased [44]. Further analysis showed that exosomal *miR-301a-3p* from ESCs reduced the phagocytic capacity of macrophages via regulating the PTEN-PI3K axis in EMs [44]. It is worth waiting for more studies to elucidate the relationship between miRNA from EVs and macrophages in endometriosis.

Sun et al. have proved that after peritoneal injection of the EVs from the uterine tissue of EMs mice, there was an increased percentage of M2-like macrophage and a decreased percentage of M1 macrophage [37]. Additionally, EVs from the uterine tissue of EMs mice decreased the phagocytes of PF macrophages to green zymosan [37]. Therefore, Ems EVs may remodel phenotypes and phagocytic ability. The diminished phagocytic capacity of macrophages contributes to the persistence of endometrial cells following menstrual blood flow, thereby playing a crucial role in the pathogenesis of EMs [96].

## 4. Macrophage-Derived EVs in the Pathogenesis of EMs

**Figure 1:**
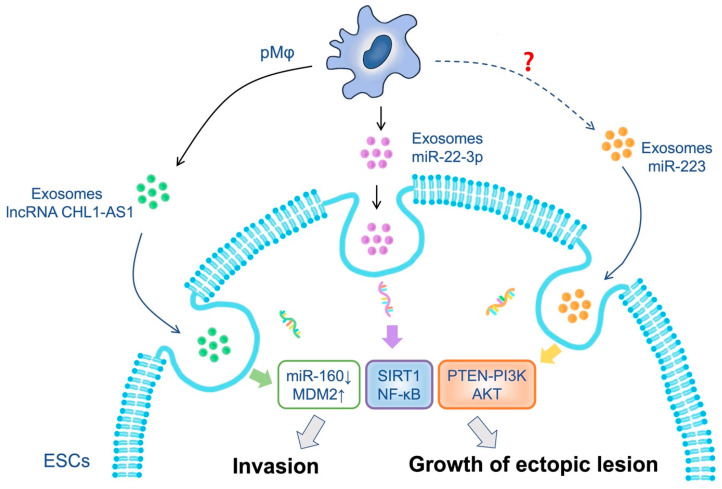
EVs from peritoneal macrophages (pMφ) in endometriosis (EMs).

Many studies have reported on the role of macrophage-derived EVs in disease development. In various diseases, macrophage-derived EVs have been found to promote cell migration, invasion, and angiogenesis [44,97,98]. In EMs patients, peritoneal macrophages are preponderant and highly active compared to healthy women [99]. EVs secreted by peritoneal macrophages are deeply involved in developing EMs (Figure 1). It has been reported that M1-derived EVs exerted a direct or indirect inhibitory effects on the migration and invasion of ESCs from EMs, and the tube formation of HUVECs [100].

Notably, lncRNA *CHL1-AS1* exhibits high expression levels in EMs [38]. MDM2, a key molecule modulated by lncRNA *CHL1-AS1*, confers promotes cell proliferation, and supports tumor growth [101]. It has been observed that the expression rate of MDM2 protein in a normal endometrium is lower than in EMs, and increased MDM2 expression leads to cell cycle dysfunction of the endometrium [102]. More interestingly, EVs-lncRNA CHL1-AS1 from peritoneal macrophages promotes the proliferation, migration, and invasion of eESCs, and restricts their apoptosis by downregulating miR-610 and upregulating MDM2 [38,103].

Specifically, *miR-22-3p* from peritoneal macrophage-derived EVs from EMs regulated the biological function of eESCs by targeting SIRT1 and activating the NF-κB signaling pathway [47]. It has been observed that the upregulation of SIRT1 assists endometrial epithelial cells in evading senescence, and promotes the occurrence of an epithelial–mesenchymal transition in EMs [104]. Therefore, this regulatory *miR-22-3p*–SIRT1/NF-κB axis should contribute to the proliferation and migration abilities and cell motility of eESCs [47].

Exosomes derived from macrophages tend to accumulate in inflammatory sites, potentially producing a proinflammatory response [105]. It has been reported that exosomal *miR-223* induced the differentiation of M2 macrophages from monocytes as well as other progenitor cell lineages that eventually mature into granulocytes or megakaryocytes, which are produced by endothelial cells, epithelial cells, and fibroblasts in the inflammatory state [106,107]. Additionally, *miR-223*, released from macrophage-derived EVs via the PTEN-PI3K/AKT pathway, promoted a malignant phenotype in epithelial ovarian cancer (EOC) cells [108]. This finding also echoed the results in EMs. Upregulation of miRNA-223 has been observed to attenuate the migratory and invasive capability of ESCs in Ems, while suppressing cell apoptosis [107]. However, the direct effect of exosomal *miR-223* on macrophages in EMs needs further elucidation.

Current studies confirm that macrophages play an important role in endometriosis. However, fewer studies about exosomes from macrophages are currently available. The specific miRNA corresponding to macrophages in EMs need more evidence to verify its functions and clinical value.

Peritoneal macrophages regulate the growth and invasion of endometrial stromal cells (ESCs) in EMs by EVs. For example, EVs deliver lncRNA CHL1-AS1 and endogenously compete to downregulate miR-160, which in turn increases MDM2 and affects the development of EMs. Exosomal miR-22-3p can activate the SIRT1/NF-κB pathway, which promotes the proliferation, migration, and invasion of ESCs. Additionally, exosomal miR-223 can also target the PTEN-PI3K-AKT axis to enhance the growth of ectopic lesions, but this still needs more research. ↑: the increased the expression; ↓: the decreased of the expression. 

## 5. Conclusions

High-level EVs can generally be found in peripheral blood, peritoneal fluid, the ectopic endometrium, and immune cells. Exosomes have been observed to deliver miRNAs, lncRNAs or proteins to regulate the proliferation, migration and invasion of ectopic lesions, inflammation, immune dysfunctions, and angiogenesis, and these processes are dependent on the EVs-mediated crosstalk between ESCs and macrophage. On the one hand, the interaction between EVs and macrophages can regulate macrophage infiltration, polarization, and phagocytosis. On the other hand, EVs derived from macrophages have been identified to affect the biological function of ectopic lesions through lncRNAs and microRNAs. However, numerous questions still need to be addressed. For example, what factors regulate macrophage-derived EVs? How do Evs modulate the function of other immune cells such as, NK cells, and T cells in the ectopic endometrium microenvironment? Investigation of these regulatory mechanisms should provide valuable insights for further developing therapeutic strategies to counter EMs.

Collectively, the exosome–macrophage axis should play an important role in the occurrence and development of EMs, which presents us with implications for the early warning, prevention, and treatment of EMs. The data of many researchers suggest that some miRNAs from serum EVs may be considered potential biomarkers for early warnings of EMs; however, the miRNAs differentially expressed by EVs in different reports, such as *miR-26b-5p*, *miR-215-5p*, *miR-6795-3p*, *miR-320a*, and *miR-22-3p*, show less conformity. As for EVs from EMs serum, there are still few studies upon which to elaborate. How we might apply the different miRNAs of EVs between EMs patients and the controls to help diagnose EMs is indeed a question that needs more significant clinical research. Therefore, multi-center clinical studies with large sample sizes are needed to further evaluate the value of EMs-specific EVs in diagnosing EMs. In addition, further research is still required to explore novel strategies for treating EMs by targeting the exosome–immune cell regulatory axis.

## Figures and Tables

**Table 2:** Functions of molecules from EVs in EMs.

Generation	Molecule	Function	Experiment	Reference
Uterine endothelial cells	miR-138/VEGF/NF-κB	Inflammation ↑	vivo and vitro	Bai et al., 2018 [60]
ESCs	lnc HOTAIR/miR-761/HDAC1/STAT3	Inflammation ↑	vivo and vitro	Zhang et al., 2022 [36]
Uterine aspirate fluid	miR-210-3P/JNK/MAPK	M1 macrophages ↓	vitro	Jiang et al., 2022 [52]
		Macrophage phagocytosis ↓		
Ectopic endometrial tissues	miR-301a-3p/PTEN/Arg-1 and PI3K	M2 macrophages ↑	vivo and vitro	Huang et al., 2022 [44]
		Macrophage phagocytosis ↓		Smith et al., 2012 [18]
ESCs	miR-214/CTGF and Collagen αI	Endometrial fibrosis ↓	vivo and vitro	Zhang et al., 2021 [61]
		M2 macrophages ↑		Peng et al., 2022 [62]
ESCs	COUP-TFII/VEGF-C	Macrophage infiltration ↑	vivo and vitro	Li et al., 2020 [63]

Abbreviations: ESC, endometrial stromal cells; VEGF, vascular endothelial growth factor; NF-κB, nuclear factor kappa B; HDAC1, histone deacetylase1; STAT3, signal transducer and activator of transcription 3; JNK, c-Jun N-terminal kinase; MAPK, mitogen-activated protein kinase; PTEN, phosphatase and tensin homolog; PI3K, phosphatidylinositol3-kinase; CTGF, connective tissue growth factor; COUP-TFII, COUP transcription factor 2. ↑: the molecules enhance the functions; ↓: the molecules suppress the functions.

## Abbreviations

CCL20	C-C motif ligand 20
CCR6	C–C chemokine receptor type 6
MAPK	mitogen-activated protein kinase
ERK	extracellular regulated protein kinases
HDAC1	histone deacetylase 1
STAT3	signal transducer and activator of transcription 3
aHIF	antisense hypoxia-inducible factor
PRDX1	peroxiredoxin-1
ANXA2	annexin A2
ITIH4	inter-a-trypsin inhibitor heavy chain H4
VEGF	vascular endothelial growth factor
TGF-β	transforming growth factor-β
GSK3B	glycogen synthase kinase 3 beta
SIRT1	silent mating-type information regulation 2 homolog-1
NF-κB	nuclear factor kappa B
IKKβ	inhibitor of nuclear factor kappa-B kinase subunit beta
PI3K	phosphoinositide-3-kinase
AKT	protein kinase B
PTEN	phosphatase and tensin homolog deleted on chromosome ten
JNK	c-Jun N-terminal kinase
CTGF	connective tissue growth factor
TAK1	TGF-β activated kinase 1
Smad7	mothers against DPP homolog 7
MDM2	mouse double minute 2 homolog
